# Outcomes in hematopoetic cell transplantation in the setting of mold infections in patients with chronic granulomatous disease

**DOI:** 10.1038/s41409-024-02389-x

**Published:** 2024-11-04

**Authors:** Ahnika Kline, Mark Parta, Jennifer Cuellar-Rodriguez, Juan Gea-Banacloche, Corin Kelly, Stefania Pittaluga, Christa S. Zerbe, Steven M. Holland, Harry L. Malech, Elizabeth M. Kang

**Affiliations:** 1 Department of Laboratory Medicine/National Institutes of Health, San Diego, CA USA; 2 Clinical Research Directorate, Frederick National Laboratory for Cancer Research, San Diego, CA USA; 3 National Institute of Allergy and Infectious Diseases, National Institutes of Health, Bethesda, Maryland USA; 4 Derpartment of Pathology, National Cancer Institute, National Institutes of Health, Bethesda, Maryland, USA

**Keywords:** Stem-cell therapies, Immunological deficiency syndromes, Fungal infection

## Abstract

Chronic granulomatous disease (CGD) is a disorder of immunity characterized by phagocyte dysfunction. Mold infections in patients with CGD are often severe and disseminated. We present patient characteristics, microbiological data, and outcomes for 26 patients with CGD who received hematopoietic cell transplantation (HCT) or gene therapy-modified cells (GT) between 2008 and 2019, with proven fungal infection either before or during their transplant. All patients engrafted, and all but one GT recipient had neutrophil recovery and evidence of functional correction. Eighteen patients (69%) are currently alive and 19 patients (73% of total, 90% of patients with repeat imaging performed) had evidence of radiographic improvement. With 3 exceptions, deaths were not principally related to the fungal infection and duration of antecedent infection did not correlate with death. *Aspergillus* species accounted for the majority of disease (50%), followed by *Phellinus* species (18%). Osteomyelitis and disseminated disease were common, as only 11 patients (42%) had disease restricted to pneumonia. Triazole therapy was used in all 26 patients, with combination therapy used in 25 (96%). HCT or gene therapy, with appropriate antifungal therapy, are viable therapies for refractory fungal infections in patients with CGD.

## Introduction

Chronic granulomatous disease (CGD) is a disease characterized as an inborn error of immunity (IEI) that predisposes patients to fungal infections, principally molds, which are often medically and surgically incurable. Correction of the immune defect by hematopoietic cell transplantation (HCT) or gene therapy (GT) alters the course of these infections. However, both the cytopenias and immunosuppression associated with these procedures have been considered a contraindication in the presence of active infection.

Incident invasive fungal infections are associated with severe morbidity and mortality in HCT for hematological disease [1]. The nature of fungal infection in CGD is profoundly different than that in hematological malignancy, as it is often chronic and even when controlled may not be considered cured. Applying diagnostic standards developed in patients who have cancer and leukemia previously led to criteria for diagnosis that were inappropriate. For instance, pre-transplant fungal disease in CGD does not present with cavitary pneumonia, which had been a diagnostic criteria for probable invasive fungal disease until the revision of the EORTC/MSG guidelines in 2020 [2, 3].

Criteria to define indications for HCT in CGD remain a work in progress, with the need to define and compare outcomes from managing the existing disease with the cost, morbidity and potential mortality of HCT. A recent report comparing short term outcomes (2.3 years) for transplantation compared to standard care in CGD was unable to identify a difference by which to clarify the decision about proceeding to transplantation [4]. The need for life-long prophylaxis against fungal disease is a consideration that is intensified by the frequently intractable nature of infection once it occurs. For these reasons, we have used HCT or lentivirus-based gene therapy (GT in gp91/X-linked CGD) in patients with active fungal infections despite historical concerns about complications during the process of transplantation in view of the limited efficacy of antifungal therapies. We describe the fungal infections prevalent or incident during CGD HCT and GT in all patients who received these procedures from 2008 to 2019, their treatment, and the response to the procedure and antifungal therapy.

## Methods

### Patient selection

Patients were all participants at the NIH with proven invasive fungal infections in protocols for HCT or GT for CGD; all provided informed consent for treatment and publication. CGD genotypes were defined by molecular methods and composed of x-linked disease due to mutations in the *CYBB* gene leading to gp91 deficiency, and autosomal recessive disease due to mutations in the *CYBA, NCF2*, and *NCF1* genes leading to p22-phox, p67-phos and p-47 deficiencies, respectively [5, 6].

Functional assessment of neutrophil function/reactive oxygen species (ROS) was performed as described with a flow cytometric assay utilizing dihydrorhodamine (DHR) [7].

### Transplant/gene therapy protocols

Patients from 5 protocols, including transplants utilizing different conditioning regimens or donors, and gene therapy, were included. Two of 5 protocol results are published and three are ongoing [8, 9]. (ClinicalTrials.gov identifiers NCT02629120, NCT03910452, NCT02234934).

The first of 5 transplant protocols used either bone marrow (BM) or peripheral blood stem cells (PBSC) for recipients with a matched related or unrelated donor (MRD/MUD). Conditioning consisted of alemtuzumab (cumulative dose 1 mg/kg) and intravenous daily busulfan (5 mg/kg, 10 mg/kg total MUD/MRD respectively) over two days, with the addition of 300 cGy total body irradiation (TBI) in MUD recipients [9]. The second protocol was for recipients with haplo-identical related donors (HRD), and utilized PBSC and conditioning with fludarabine, low dose cyclophosphamide, intravenous daily busulfan for 3 days (target area under the curve [AUC] 12,000 uMol *min or 49 mg*h/L), TBI 200 cGy, and high dose post-transplant cyclophosphamide (PTCy) 50 mg/kg/d for 2 days [8]. The third protocol was a modification of the first, with identical conditioning, but used high dose PBSC (maximum 10 × 10^6^ CD34+ cells/kg recipient for MRD/MUD recipients with the addition of PTCy for GVHD prophylaxis. The fourth transplant protocol is for recipients of HRD products. Conditioning consists of alemtuzumab given in distal (day -21 to -17) and proximal (day -6) doses, intravenous busulfan 3.0 mg/kg per day for 3 days, 200 cGy and PTCy and sirolimus GVHD prophylaxis. Patients with C-reactive proteins (CRP) > 100 mg/L are excluded. In a fifth protocol, X-linked CGD patients received autologous hematopoetic cells (enriched for CD34+ stem cells) transduced with the G1XCGD lentiviral vector (pCCL ChimGp91/VSVg lentiviral vector) [10]. Conditioning was with IV busulfan twice daily for 3 days (target AUC of 65–70 mg*h/L).

At the time of this analysis, 67 total patients had been enrolled in the bone marrow transplant protocols above.

Identification of fungi was traditional morphologic criteria, aided by molecular diagnostics (sequencing) and matrix-assisted laser desorption/ionization-time of flight (MALDI-TOF) analysis where available. Antifungal susceptibility testing was performed at the University of Texas Health San Antonio Fungus Testing Laboratory according to approved methodologies. Breakpoints for molds based on mean inhibitory concentration (MIC) are generally lacking, with the exception of voriconazole for *Aspergillus fumigatus*, for which an MIC of less than or equal to 0.5 µg/ml is considered susceptible, 1 µg/m is considered intermediate, and greater than or equal to 2 µg/ml is considered resistant [11].

Statistical testing comparing outcomes in the patients who died to the living patients was performed using a Mann-Whitney nonparametric test with significance being likely if *p* < 0.01.

### Clinical and disease characteristics

Day 0 was the day of cell infusion, with negative (−) and positive (+) indicating the days prior or post infusion, respectively. Neutropenia was a neutrophil count of <500 cells/µl. Fever was defined as a temperature of 38 °C or greater.

Fungal infections were characterized by the time of onset and response to medical therapy. Time of onset was defined as initial radiologic finding and/or microbiologic diagnosis. Incident disease was not diagnosed prior to transplant/gene therapy. The disease was stable if the patient had no clinical symptoms related to the disease and pre-transplant/gene therapy radiology revealed no changes to suggest progression at day 0. A patient was determined to have progressive infection if they continued to present signs or symptoms of disease and/or showed radiographic changes suggestive of more or new organ involvement at day 0.

The risk of an outcome related to the fungal infection was assessed by the duration of neutropenia in days, the presence of graft-versus-host disease (GVHD) (almost always a corticosteroid-requiring complication) and/or the use of corticosteroids in the absence of a diagnosis of GVHD, often for engraftment syndrome [12–14].

Outcomes included overall survival, attributable death (determined by chart review by the investigators) engraftment, and response of the fungal infection. Analysis of the response of the fungal infection to HCT was based on clinical symptoms and radiographic evaluation by computed tomography (CT) and positron-emission tomography (PET) scans.

Sufficient drug level for therapy is defined as voriconazole trough greater than 1 mg/L or a posaconazole trough of greater than 1 mg/L or an itraconazole trough greater than 0.5 mg/L or an isavuconazole level of greater than 1 mg/L [15–18].

Patients with CGD managed at the National Institutes of Health receive a prophylactic mold-active azole regimen against fungal infections, and all patients received antifungal prophylaxis or treatment at the time of transplant/GT.

## Results

### Patients

Median patient age at transplant/GT was 18 years old (IQR 11-27). Twenty-three of 26 patients were male (88%), and of these male patients, 20 had X-linked CGD. Of the AR CGD patients, four had p47-phox deficient CGD, one had p22-phox deficiency and one had p67-phox deficiency. (Table 1).

**Table 1:** Patient demographics.

Patient Number	Year	Age (at transplant)	Sex	Type of CGD^a^	Type of transplant^b^	Protocol	Type of Fungal Disease	Anatomy	Years of Infection Prior to Transplant
1	2005-10	17	F	p47 AR	MUD	1	stable	Pneumonia	3
2	2005-10	8	M	XL	MUD	1	progressive	Pneumonia, thoracic spine osteomyelitis	3
3	2005-10	18	M	p47 AR	MUD	1	progressive	Pneumonia	5
4	2005-10	6	M	XL	MRD	1	progressive	Pneumonia, osteomyelitis	0.5
5	2011-15	18	M	XL	MUD	1	progressive	Liver abscess, biliary obstruction	1
6	2011-15	11	M	XL	MUD	1	stable	Pneumonia	1
7	2011-15	31	M	XL	MUD	1	stable	Pneumonia, vertebral osteomyelitis	1
8	2011-15	5	M	XL	MUD	1	stable then incident^b^	Disseminated, CNS	5
9	2011-15	9	M	XL	MUD	1	progressive	Paraspinal abscess	1.5
10	2011-15	20	M	XL	MUD	1	progressive	Pneumonia, rib/spine osteomyelitis	10
11	2011-15	18	M	XL	HRD	2	stable	Pneumonia	1
12	2016-19	6	M	XL	MUD	3	incident	Nodular pneumonia	N/A
13	2016-19	26	F	p67 AR	HRD	2	progressive	Pneumonia, sternal osteomyelitis	12
14	2016-19	18	M	XL	HRD	2	incident	Pneumonia	N/A
15	2016-19	16	M	XL	HRD	2	incident	Nodular pneumonia	N/A
16	2016-19	25	M	XL	MUD	3	progressive	Pneumonia, foot lesion	0.5
17	2016-19	24	M	XL	GT	5	progressive	Nodular pneumonia	0.5
18	2016-19	3	M	XL	GT	5	progressive	Nodular pneumonia, cerebellum, spine/iliac osteomyelitis	1
19	2016-19	34	M	XL	MUD	3	progressive	Pneumonia, thoracic spine osteomyelitis, abdominal and brain abscesses	14
20	2016-19	30	M	XL	MRD	3	progressive	Right thigh muscle	6
21	2016-19	48	M	p47 AR	MUD	3	stable	Pneumonia	1
22	2016-19	31	M	XL	GT	5	stable	Paraspinal, vertebral osteomyelitis	12
23	2016-19	43	M	XL	MUD	3	incident	Nodular pneumonia	N/A
24	2016-19	13	F	p22 AR	MUD	3	progressive	Pulmonary, mediastinum/rib/spine osteomyelitis, liver	11
25	2016-19	16	M	XL	HRD	4	stable	Left upper lobe pneumonia	2
26	2016-19	27	M	p47 AR	MUD	3	stable	Liver and omentum	1

*MRD* matched related donor, *MUD* matched unrelated donor, *HRD* Haplo-identical related donor, *GT* lentiviral-based gene therapy.

^a^XL, X-linked disease (gp91 deficiency), and autosomal recessive (AR) disease (p22-phox, p67-phox, and p47-phox deficiencies).

^b^Disease was stable until transplant, then worsened post-transplant.

### Characteristics and microbiology of fungal infections

Twenty-six patients with CGD had either stable/progressive fungal infection or developed a fungal infection post-HCT. All patients had proven fungal disease consistent with current definitions with radiographic findings suggestive of fungal infection in CGD [2]. Of the 26, 13 had progressive disease before the time of transplant, 8 had stable disease, and 5 had incident disease during/after HCT. For patients with known infection prior to HCT/GT, (21/26), the median duration of infection was 1.75 years (IQR 1–6 years) [1–6] (Table 1).

*Aspergillus* species (14/26) were most common, specifically *Aspergillus fumigatus* (4) and A. *nidulans* (4). The 2nd most common organism was *Phellinus* sp. (5) followed by *Scedosporium apiospermum* (2). Incident infection after HCT was most often in the form of nodular pneumonia and was caused by *A. fumigatus*, *A. niger*, *Rhizopus* sp., and unknown (positive pathology/negative microbiology).

Pneumonia, including nodular pneumonia, was the most common manifestation of fungal infection (20/26). However, of patients with pneumonia, 45% (9/20) also had osteomyelitis due to direct extension to the thoracic spine and/or ribs. Only eleven patients had isolated pneumonia. Three patients had pre-existing central nervous system (CNS) disease, of whom all are now deceased. Three patients had liver involvement (Tables 2 and 3).

**Table 2:** Transplant outcomes.

Patient	Antifungal	Therapy changed?	Duration of Neutropenia (days)	GVHD	Granulocytes	Radiology Improved?	Days to death	Outcome
1	PO VRC	No change	17	No	No	N/A	N/A	Success
2	Liquid POSA, IV VRC, echino	No change	10	No	Yes	Yes	N/A	Success
3	Liquid POSA, PO then IV VRC	Added AMB and echino	44	No	No	No	663	Death (Evan’s syndrome, infection recurrence)
4	IV VRC, echino, TRB	No change	8	N/A	Yes	Yes	N/A	Success
5	IV VRC, echino, AMB	No change	8	Yes	Yes	N/A	91	Death (alveolar hemorrhage)
6	Liquid POSA, Po then IV VRC (1 dose IV only)	Added AMB and echino	14	Yes	No	No^b^	N/A	Success
7	PO then IV VRC, echino	No change	17	No	Yes	Yes	N/A	Success
8	ITC and VRC IV	Added AMB and echino	19	Yes	No	Yes	296	Death (status epilepticus, CNS Aspergillosis)
9	Liquid POSA	No change	10	No	No	Yes	N/A	Success
10	PO POSA, echino, AMB, TRB	No change	4	No	Yes	Yes	N/A	Success
11	PO then IV POSA, echino, TRB	Added AMB	21	Yes	Yes	Yes	N/A	Success
12	FLC	Added IV POSAA, AMB and echino	16	No	No	Yes	N/A	Success
13	IV POSA, echino, TRB	No change	23	Yes	Yes	Yes	N/A	Success
14	ITC	Added AMB and echino, changed to POSA liquid then PO then IV	21	Yes	No	N/A	316	Death (alveolar hemorrhage, liver GVHD)
15	PO VRC, echino	Changed to IV then liquid VRC	12	Yes	No	Yes^c^	N/A	Success
16	PO POSA, echino, TRB	No change	14	No	No^a^	Yes	N/A	Success
17	Liquid POSA, ISA	Added TRB	2	No	No	Yes	N/A	Success
18	IV POSA, echino, AMB, TRB	No change	18	No	Yes	N/A	44	Death (autoimmune thrombocytopenia, intracranial hemorrhage)
19	IV then PO then IV ISA, echino, TRB	No change	9	N/A	Yes	Yes	25	Death (engraftment syndrome, TRALI)
20	IV AMB	Added IV POSA	14	N/A	Yes	N/A	39	Death (engraftment syndrome)
21	PO POSA	No change	15	No	No	Yes	N/A	Success
22	IV then PO ISA, PO then IV POSA, TRB	No change	3	No	No	Yes	N/A	Success
23	PO then IV POSA	Added AMB and echino	23	Yes	No	Yes	N/A	Success
24	PO POSA, ISA, echino, TRB	No change	13	No	No	Yes	N/A	Success
25	PO then IV POSA, echino, TRB	No change	26	No	No	Yes	N/A	Success
26	PO POSA, echino, TRB	Changed to liquid then IV POSA	25	No	No	Yes	263	Death (sepsis and liver aspergillosis)

*ISA* Isavuconazole, *echino* Echinocandin, *CAS* Caspofungin, *MFG* Micafungin, *BDG* Beta-D-glucan, *Asp Ag* Aspergillus Antigen.

^a^patient received granulocytes prior to transplant only;

^b^not markedly abnormal prior to transplant or after;

^c^worsened then improved at 2 y follow up.

**Table 3:** Isolate data.

Patient	Isolate	AMB MIC	VRC MIC	POS MIC	ISA MIC	CAS MIC	MFG MIC	TRB MIC	Max BDG	Max Asp Ag
1	*Theilavia spp.*^a^	2	0.25	0.25	N/A	4	N/A	N/A	Neg	Neg
2	*Aspergillus nidulans*	0.5	0.5	0.25	N/A	N/A	N/A	0.25	>500 pg/ml	N/A
3	*Aspergillus terreus*	4	0.5	0.25	N/A	1	<=0.015	0.25	>500 pg/ml	5.59
4	*Aspergillus nidulans*	1	0.25	0.125	N/A	2	N/A	0.125	289 pg/ml	Neg
5	*Pyrenochaeta romeroi*	0.5	1	0.5	N/A	N/A	4	N/A	343 pg/ml	Neg
6	*Aspergillus fumigatus (OSH)*	N/A	N/A	N/A	N/A	N/A	N/A	N/A	N/A	N/A
7	*Scedosporium apiospermum*	N/A	1	2	N/A	2	0.25	N/A	31 pg/ml	N/A
8	*Aspergillus spp. (OSH)*	N/A	N/A	N/A	N/A	N/A	N/A	N/A	N/A	Neg
9	*Phellinus spp*.	0.25	<=0.03	<=0.03	N/A	>8	>8	>2	Neg	N/A
10	*Aspergillus nidulans*	>2	0.125	0.5	N/A	N/A	0.06	0.125	N/A	Neg
11	*Scedosporium apiospermum*	2	1	2	2	2	1	>2	455 pg/ml	N/A
12	*N/A (hyphae on pathology)*	N/A	N/A	N/A	N/A	N/A	N/A	N/A	N/A	Neg
13	*Aspergillus nidulans*	1	0.25	0.5	0.25	N/A	N/A	0.06	N/A	3.61
14	*Rhizopus spp*.	0.25	N/A	0.25	0.25	N/A	N/A	>2	>500 pg/ml	Neg
15	*Aspergillus fumigatus*	2	1	0.25	1	N/A	<=0.015	N/A	N/A	Neg
16	*Aspergillus pseudoviridinutans*	1	4	1	4	0.13	N/A	N/A	N/A	Neg
17	*Phellinus spp*.	0.06	0.5	0.125	0.06	N/A	N/A	>2	N/A	Neg
18	*Aspergillus fumisynnematus*	1	1	0.06	0.5	<=0.015	>8	0.125	Neg	Neg
19	*Phellinus spp*.	0.25	1	1	N/A	>16	N/A	N/A	Neg	Neg
20	*Phellinus spp*.	0.5	<=0.03	<=0.03	<=0.03	N/A	N/A	N/A	N/A	Neg
21	*Corprinellus and Oxyporus spp*.	N/A	N/A	N/A	N/A	N/A	N/A	N/A	59 pg/ml	Neg
22	*Phellinus spp*.	0.13	0.25	0.03	N/A	16	N/A	1	N/A	Neg
23	*Aspergillus niger*	N/A	N/A	N/A	N/A	N/A	N/A	N/A	N/A	Neg
24	*Aspergillus fumigatus (OSH)*	N/A	N/A	N/A	N/A	N/A	N/A	N/A	N/A	Neg
25	*Polyporales spp*.	0.25	0.125	0.06	0.06	N/A	>8	>2	N/A	N/A
25	*Aspergillus fumigatus*	1	0.5	0.25	1	N/A	<=0.015	0.5	N/A	N/A
26	*Aspergillus felis*	1	4	0.5	4	0.03	<=0.015	N/A	N/A	Neg

*ISA* Isavuconazole, *echino* Echinocandin, *CAS* Caspofungin, *MFG* Micafungin, *BDG* Beta-D-glucan, *Asp Ag* Aspergillus Antigen.

^a^Isolated from new pulmonary lesion prior to transplant.

### Treatment prior to and during HCT/GT

All but one patient were on an azole antifungal at the time of HCT/GT, and 24 were receiving a newer triazole (one patient was on fluconazole only). Nine patients had an escalation of therapy after induction with the most common change being the addition of liposomal amphotericin B (LAmB) (7 patients), plus or minus an echinocandin (5 patients). Many patients were on combination antifungal therapy. Including escalations, 9 of 26 patients were on triple drug therapy with an azole, echinocandin and LAmB. An additional 10 patients were on combination therapy with an azole and echinocandin, with 8 of these patients also receiving terbinafine. The median duration of antifungal therapy after HCT/GT was 8 months (IQR 5–13).

### Clinical outcomes

The median duration of neutropenia was 14.5 days (IQR 10-21). All patients eventually engrafted/recovered from neutropenia. Eighteen of 26 (69.2%) patients remain alive as of 2021. There was no statistically significant difference in the duration of neutropenia between deceased and surviving patients, despite a trend toward longer neutropenia in the deceased (mean difference 5.97 days, median difference 4.5 days). (Table 2).

Eight patients died. The median time to death was 177 days (IQR 41.5–356). Contributing causes of death included diffuse alveolar hemorrhage/transfusion-related acute lung injury (TRALI)(3 patients), engraftment syndrome (2 patients), and autoimmune thrombocytopenia (2 patients). Four patients had evidence of recurrent or persistent infection at the time of death. There was no significant difference in duration of antecedent infection between patients who died and those who survived.

### Attributable deaths

Patient 18 was a 3-year-old boy who died of intracranial hemorrhage during profound anti-platelet antibody associated thrombocytopenia, perhaps related to conditioning for GT, in the context of presumed prior CNS fungal disease. Patient 8 was a 5-year-old boy who died of cerebral aspergillosis during corticosteroid therapy for GVHD. Patient 26 was a 27-year-old man with chronic *A. felis* infection [19]. Despite radiographic improvement prior to transplant and early during the post-transplant period and strong myeloid engraftment (74% at 3 months), severe systemic inflammatory disease consistent with his pre-transplant condition occurred and viable *A. felis* was recovered from a liver biopsy at 3 months post-transplant. He died 9 months post-transplant with presumed progression of fungal disease.

### Ancillary therapy and non-infectious events

Ten patients received granulocyte transfusions after transplantation, and an eleventh patient received them before transplantation, but sensitization precluded use during the transplant. There were 4 deaths (40%) in patients who received granulocytes after transplantation compared to 4 deaths (27%) in those who did not.

GVHD occurred in 8 of 23 patients who could be assessed (35%), and three of the 8 died. Seven of 8 patients (88%) with GVHD received corticosteroids, as did six of fifteen patients (40%) without GVHD. Tocilizumab was given to four patients, three for engraftment syndrome and one for elevated inflammatory markers during and after the transplant). (Table 2).

### Radiographic evaluation

**Fig. 1:**
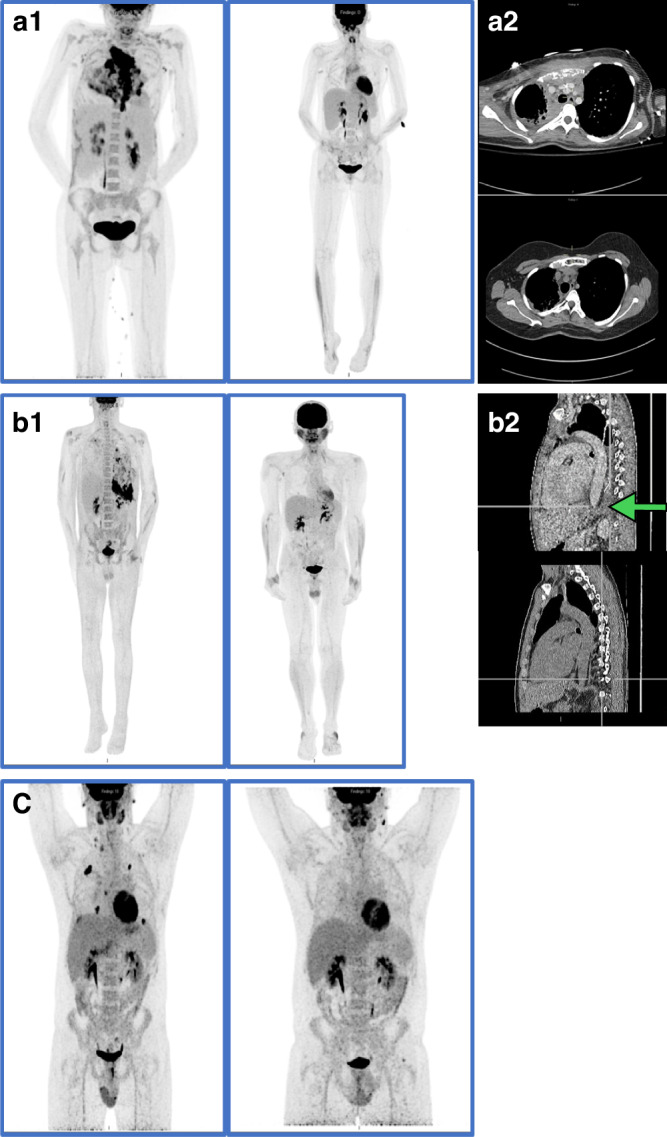
Representative radiology (left to right). **a** Patient 13. **a1** PET scans: 5 weeks prior to transplant (left), and 1 year post transplant (right). **a2** CT scans. 5 weeks prior to transplant and (left) 3 years post transplant (right) **b** Patient 16. **b1** PET scans: 10 weeks prior to transplant (left), and 1 year post transplant (right). **b2** CT scans. 10 months post transplant (right), two months post transplant(left). Arrow indicates area of infection. **c** Patient 22. PET scans: 20 months prior to gene therapy (left), and 3 months post gene therapy (right).

Nineteen patients had radiographic improvement (as defined by either a reduction in lesion size seen on CT, magnetic resonance imaging (MRI) or PET scan at 6 months or more after transplant) (Fig. 1). Of the remaining seven patients, four died before having post-procedure imaging. There were 2 patients with unchanged imaging with no evidence of clinical progression (Table 2).

**Fig. 2:**
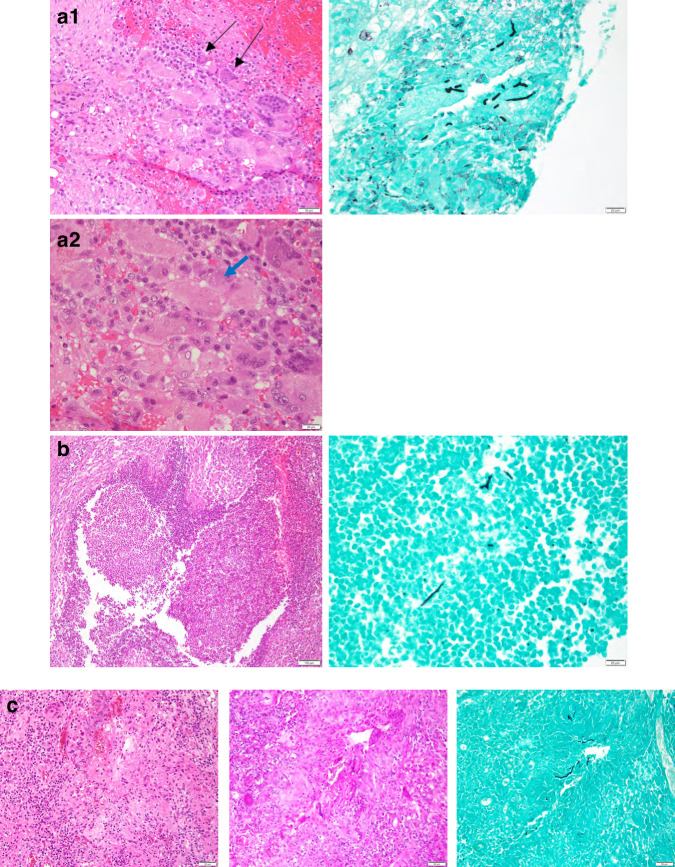
Representative pathology imaging from 3 patients. **a** Patient 13, 1 month prior to transplant. Soft tissue with granulation tissue with clusters of multinucleated giant cells and fungal forms present within giant cells (black arrows delineate giant cells, blue arrow shows fungal forms) **a1** H&E (left) at 200x, GMS (right) at 400x, **a2** H&E (below) at 400x **b** Patient 16, 3 months prior to transplant. Left upper lobe wedge. Marked granulomatous inflammation with neutrophilic abscess formation. Rare fungal forms are present within the abscess H&E (left) at 100x, GMS (right) at 400x **c** Patient 22, 3 years prior to gene therapy. Epidural lesion. Fibrous tissue with a dense lymphoplasmacytic infiltrate, multinucleated giant cells and rare fungal forms present. H&E (left), PAS (middle), GMS (right) at 200x.

Three representative cases demonstrate the complexity of radiographic evaluation, and the difficulties in categorizing patient outcomes. Patient 13 (AR p67-phox deficient CGD) is a 26 year-old woman transplanted with a 12 year history of progressive *A. nidulans* pneumonia and osteomyelitis with destruction of the sternum. With full engraftment after HRD HCT, the radiographic followup showed persistence of scar and chronic pulmonary parenchymal damage, but 4 years post-HCT there was evidence not only of stability of the pulmonary and bone radiology, but remineralization of the ground substance of the sternum (Fig. 1. A1, A2). Patient 16, a 25 year-old man with gp91phox-deficient (X-linked) CGD, was transplanted for chronic *Aspergillus pseudoviridinutans* infection. The disease characteristically included primary lung disease that extended to the spine and then below the diaphragm (Fig.1, b1, b2). He fully engrafted by 3 weeks post-HCT and radiology 1 year post-HCT showed resolution of reactivity on PET scan and regression of disease (Fig. 1, b2). Patient 22 received GT at age 31 years in the context of chronic/recurrent *Phellinus* sp. pneumonia and osteomyelitis. Post-GT immunological evaluation yielded DHR activity improvement from < 10% at baseline to >70% by 2 months post-GT. PET imaging performed at 3 months post-GT demonstrated resolution of the inflammatory pulmonary disease (Fig. 1. c1, c2). For reference and elucidation of the histopathological nature of the disease process in CGD, and to differentiate this from that seen with invasive aspergillosis in hematological/malignant disease, Fig. 2 includes representative pre-therapy granulomatous inflammation and fungal forms for these 3 patients.

## Discussion

We provide evidence that active and progressive fungal infections in CGD do not preclude the successful achievement of HCT or GT, and show significent evidence for infection improvement or cure. In 2015, we reported successful transplantation of a boy with CGD and serious *Scedosporium* infection [20]. In our 2017 report on allogeneic reduced-intensity conditioning HCT, 7 reciptients had active fungal infections at the time of transplantation [9]. A set of 3 children with CGD transplanted for refractory fungal infections has been reported subsequenty from Europe [21]. While current literature focuses on the stage of disease resolution prior to transplantation, there is no literature that encompasses HCT/GT as a modality for the treatment of refractory fungal infection. Two prior studies (a registry study from 2017 and a single institution experience reported in 2021) have examined outcomes in patients with fungal infections prior to transplantation [22, 23]. These reports support transplantation in patients with resolved or low-to-moderate burden fungal infection assessed by radiographic criteria. These are not comparable populations to that reported here as the primary goal is to cure the cancer in patients with malignancy.

An older literature had assessed the outcomes of HCT in hemotological disease patients with prior fungal infection, but the analysis is appropriate to a disease process that is different than that described here, particularly to the degree that we transplanted patients in order to cure fungal infections. With a small proportion of patients coming to HCT with prior invasive aspergillosis (1.9%), 29% had post-transplant recurrence, and this group had decreased survival and higher transplant-related mortality (TRM). With very small numbers, these differences were not found when the patients had >1 month of pre-HCT treatment and resolution of radiographic abnormalities [24]. In the setting of HCT after proven or probable aspergillosis, an adequate duration of therapy and radiographic resolution of the findings associated with invasive aspergillosis as determinants of decreased risk of adverse outcomes during HCT suggest that there is some degree of functional “cure” achievable. A report of cancer patients transplanted with antecedent fungal infection, published in 2006, found that prolonged neutropenia was associated with aspergillosis progression, as was the state of the underlying disease and the interval from systemic therapy to HCT [25]. We did not replicate a relationship between duration of neutropenia and transplant outcome in our cohort.

The survival in this report of 69.2% is lower than that seen in our large series of complete followup for a protocol of HCT with MRD/MUD products (82.5% survival) with no statistically significant metrics associated with mortality. All of the transplant recipients in this report engrafted and GT recipients recovered from neutropenia, and duration of neutropenia was not associated with survival. This is consistent with our published reports of completed protocols for MRD/MUD and HRD transplantation (mean duration neutropenia 13.5 and 19 days, respectively) [8, 9]. It is notable that all three patients with pre-existing CNS disease died, even though one had a cause of death not attributable to infection (Patient 19). The three deaths associated with persistent fungal disease in this series do suggest that some aspects of unsuccessful transplantation lead to a final common pathway of hyperinflammation, with CNS hemorrhage associated with autoimmune thrombocytopenia, progression of CNS infection in the context of immunosuppressive therapy for GVHD, and a transplant that was unsuccessful in controlling the inflammatory state post-transplant, which led to multiorgan failure and persistent infection.

Mortality was 40% in patients with GVHD compared to 20% of those without, but attributing significance is limited by the low incidence of GHVD in this cohort (31%). In the two series we have published that provide data for comparison, rates of GVHD were 45 and 100% in the MRD/MUD and HRD protocols, respectively. This should be seen in the context of a very small HRD protocol (7 recipients) in which corticosteroid refractory GVHD became a stopping criterion for the study, with 2 deaths not attributable to progressive infection [8, 9]. Ongoing protocols in GT and MRD/MUD and HRD HCT are not complete and therefore data are not available.

Fungal microbiology may contribute to infection presentation and progression in CGD, before and during therapy. There is an outsized representation here of pre-HCT/GT basidiomycete infection, which has been previously reported [26, 27]. Understanding the importance of an individual *Aspergillus* species to CGD outcomes is extraordinarily difficult due to changes in taxonomy [28]. We also cannot demonstrate a relationship of antifungal susceptibilities to outcome. Like previous investigations, we cannot demonstrate the relevance of a specific fungal pathogen to outcome, but this is confounded by the use of multiple protocols, differences in post-transplant immunosuppression, and a lack of uniform physiology in CGD [29, 30].

We believe that multidrug antifungal therapy is essential for successful transplant outcomes in patients with CGD. Single drug therapy for invasive aspergillosis yields unsatisfactory results often and the utility of combination therapy is debated [31–35]. There is temporal heterogeneity in the type and route of triazole administration in these patients. Although we have recently used posaconazole as a preferred agent, evidentiary validation for posaconazole for aspergillosis has only recently appeared [33]. The post-HCT/GT duration of therapy was determined case by case, with little support from literature [36]. Although shortening post-transplant therapy is a goal, all 8 patients who died were on antifungal therapy until death (data not shown). We commonly use terbinafine, which only recently has been reported as a potentially effective agent in combination therapy [37, 38]. We have learned that therapeutic index, ease of administration and reliable pharmacokinetics are the major considerations when the fungal and patient variables are so great and difficult to characterize. With a dominant successful effect of combination therapies without amphotericin B, that agent has receded in use and its toxicity rarely seems justified.

We applied surrogate markers such as the serum ß-d-glucan or serum aspergillus antigen (galactomannan) inconsistently, since our cases are dominated by microbiological and histological evidence and radiology is a more useful index of extent of disease [39]. In our patient cohort, all but 5 patients had a galactomannan checked and it was positive in only 2, despite 14 *Aspergillus* infections (sensitivity 14%). Serum ß-d-glucan had greater sensitivity (positive in 2/3 of the cases in which it was checked) but its lack of specificity precludes its utility [40]. In these patients, a negative galactomannan is not useful, nor is a positive serum ß-d-glucan.

A critical factor in achieving an acceptable outcome is the characterization of disease prior to HCT/GT. Based on our assessment of the disease process prior to HCT, we believe that excessive inflammatory disease (a C-reactive protein > 100 mg/L in the month prior to conditioning) represents an unacceptable risk for an adverse outcome related to the HCT, but not necessarily in relation to the fungal infection with the possible exception of CNS disease. The role of the transplant platform and the response to therapy of the infection are important considerations that are not yet understood. Inflammation in CGD is not limited to that caused by infection [41].

There are limitations to this investigation. Because the study spans more than a decade, there is also a differential amount of follow up. Our treatment approaches have changed through the years. Granulocyte transfusions lack any substantial evidentiary basis in this context. Together with their potential immediate toxicity and risk of sensitization that might increase the risk of rejection, we no longer use granulocyte infusions to lessen the duration of neutropenia in our patients. As noted above, patient 16 was broadly sensitized by granulocyte infusions, had a progressive and aggressive infection, and had a completely acceptable outcome without granulocyte support during transplant. We also do not routinely perform surgery to debulk fungal infection, another intervention for which there is no evidentiary validation, and in our series, as detailed above, patient 13 failed multiple rounds of debulking and with substantial disease present at transplant, was cured by the transplant. Lastly, this paper examines only fungal infections, but pre-existing/chronic bacterial infections were frequent, as well as other manifestations of CGD such as colitis. The contribution of the other types of infections to therapeutic outcomes has been addressed in another report [42]. The general trend in reporting outcomes of transplantation in CGD reinforces adverse outcomes for older recipients, median age here 18 years as opposed to 7 years of age in a large European cohort [43].

In summary, through multiple variables including patient genotype, infecting organism and transplant platform or gene therapy, the majority of our patients with fungal infections tolerated the therapeutic procedure with few complications related to the infection. Followup demonstrated both clinical and radiographic resolution in the majority of cases. Procedure-related characteristics and complications (cytopenias, GVHD and inflammatory disease) may have contributed to poor outcomes in a few cases, but the majority of patients had a successful transplant and infection response. Further investigation is necessary to determine whether predictive tools can be developed to determine which patients will ultimately have poor outcomes. Mold-active triazole therapy, often in combination with other agents, is an important part of successful transplantation. A more rational approach to therapy would be facilitated by better microbiologic characterization of fungi and their responses to drugs, a correlation of in vitro studies to outcomes, and a better understanding of post-transplant immunological correction, which is ultimately essential for the complete cure of fungal infection.

## Data Availability

The data that support the findings of this study are not openly available due to reasons of sensitivity and are available from the corresponding author upon reasonable request. Data are located in controlled access data storage at the National Institutes of Health.
